# 2-Methyl­imidazolium tetra­kis­(2-thenoyltri­fluoroacetonato-κ^2^*O*,*O*′)neodymium(III)

**DOI:** 10.1107/S2414314625005693

**Published:** 2025-06-27

**Authors:** José Manuel Bravo-Arredondo, Sylvain Bernès, Karen Mejía, Bertin Anzaldo, Erick Ramírez, David Moro

**Affiliations:** a Posgrado en Dispositivos Semiconductores, Benemérita Universidad Autónoma de Puebla, Prolongación 14 Sur, IC5, 72570 Puebla, Pue., Mexico; b Facultad de Ciencias Básicas Ingeniería y Tecnología Universidad Autónoma de Tlaxcala, De Apizaquito 20 de Noviembre 90401 Apizaco Tlax Mexico; c Instituto de Física Luis Rivera Terrazas, Benemérita Universidad Autónoma de Puebla, Av. San Claudio y 18 Sur, 72570 Puebla, Pue., Mexico; d Centro de Química del Instituto de Ciencias, Benemérita Universidad Autónoma de Puebla, IC8, C.U., San Manuel, 72570 Puebla, Pue., Mexico; e Facultad de Ciencias Químicas, Benemérita Universidad Autónoma de Puebla, Av. San Claudio y 18 Sur, 72570 Puebla, Pue., Mexico; f Instituto de Ciencias, Benemérita Universidad Autónoma de Puebla, Av. San Claudio y 18 Sur, 72570 Puebla, Pue., Mexico; Vienna University of Technology, Austria

**Keywords:** crystal structure, neodymium, 2-thenoyltri­fluoro­acetone, disorder, hydrogen bonds

## Abstract

The title complex, (C_4_H_7_N_2_)[Nd(C_8_H_4_F_3_O_2_S)_4_], is a salt formed *via* the acid–base reaction between 2-thenoyltri­fluoro­acetone (HTTA) and 2-methyl­imidazole (MeIm) in presence of neodymium(III) tri­fluoro­methane­sulfonate. The resulting compound features [Nd(TTA)_4_]^−^ anions with the central rare-earth metal cation placed on a twofold rotation axis, and [MeImH]^+^ cations, disordered over an inversion centre. Cations and anions are linked through N—H⋯O hydrogen bonds to form zigzag chains running along [001].

## Abstract 2

The title neodymium salt includes anions and cations linked by N—H⋯O hydrogen bonds to form zigzag chains extending along [001] in the crystal.

## Graphical abstract

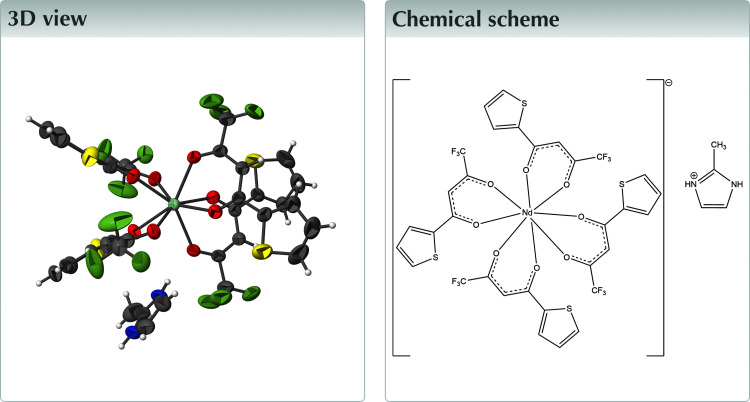

## Structure description

2-Thenoyltri­fluoro­acetone (HTTA) is a *β*-diketone ligand with chelating ability for rare-earth ions that can be used to produce highly luminescent complexes of Eu^3+^, Tb^3+^ and Sm^3+^, with high quantum yields and long luminescence lifetimes, because non-radiative decay pathways are minimized in the rigid coordination environment (Gujar *et al.*, 2019 ▸). The ligand is also able to sensitize lanthanide-centred emission *via* an efficient antenna effect. Nd^3+^ has sharp and well-defined *f–f* transitions, which makes it ideal for near-infrared luminescence applications. By coordinating this metal with *β*-diketonates like TTA^−^, one can expect the development of more efficient luminescent materials, with practical applications in optoelectronics, catalysts and biomedicine (Ahmed *et al.*, 2020 ▸).

**Figure 1:**
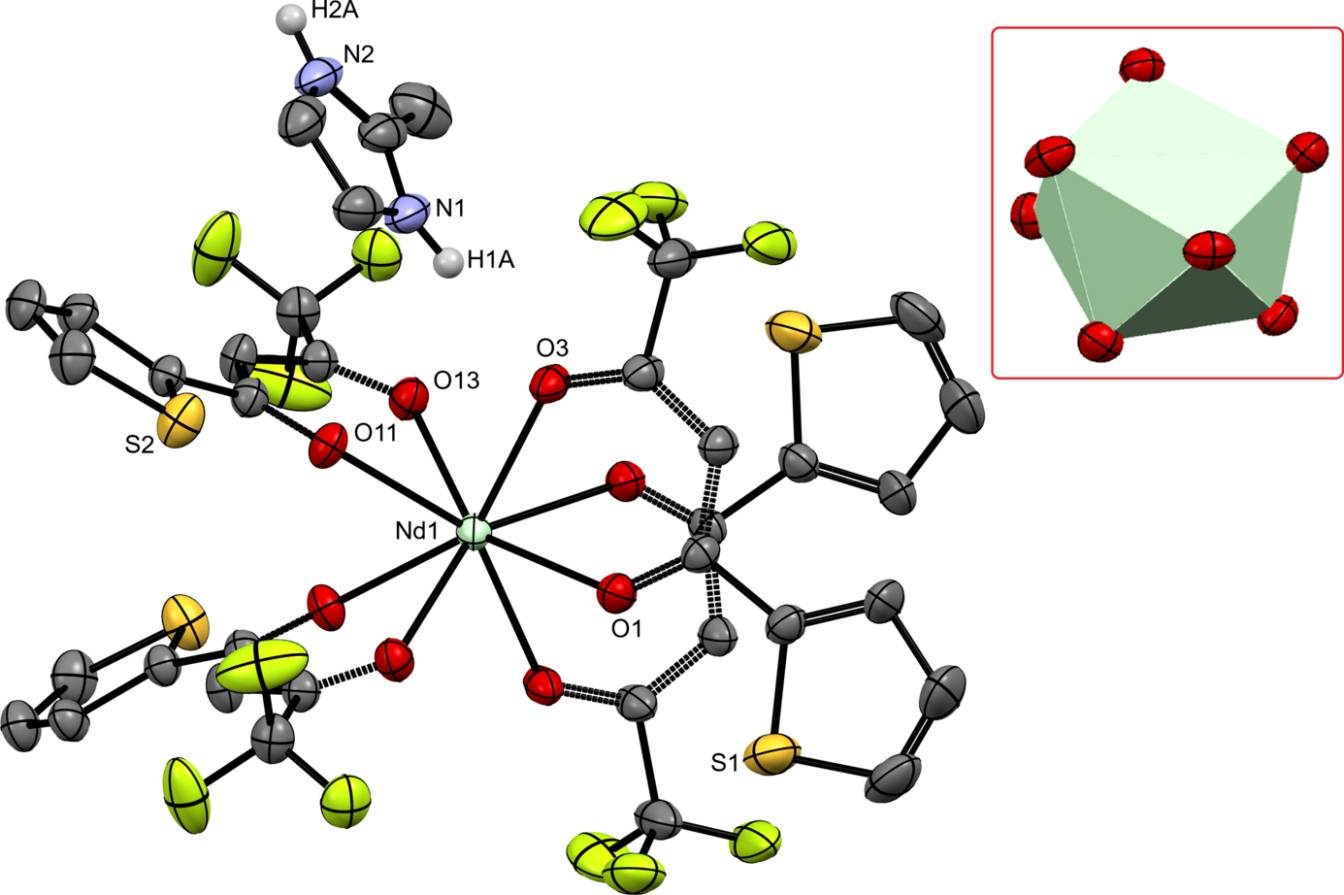
Structure of the title compound, with displacement ellipsoids drawn at the 30% probability level. Only one disordered cation is shown for clarity. Non-labelled S and O atoms are generated by symmetry operation 1 − *x*, *y*, 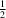 − *z*. Only H atoms belonging to the NH groups in the cation are given. The anti­prismatic coordination polyhedron around Nd1 is represented in the inset.

In this context, we synthesized the title compound, [MeImH][Nd(TTA)_4_] using MeIm (2-methyl­imidazole), HTTA, and neodymium(III) tri­fluoro­methane­sulfonate as starting materials. During the sonochemical reaction, an acid–base reaction occurs between MeIm (p*K_b_* = 6.1) and HTTA (p*K_a_* = 7.4) to form the title salt. The anion is placed on the twofold rotation axis in space group *C*2/*c*, while the cation is disordered over an inversion centre, with occupancy fixed to 1/2 in the asymmetric unit. The central ion Nd^3+^ forms a slightly distorted square-anti­prismatic polyhedron with eight O atoms of the TTA^−^ ligands (Fig. 1 ▸). The distortion is reflected in Nd—O bond lengths in the range 2.396 (2) to 2.4758 (18) Å, which are comparable to bond lengths observed in other salts of the [Nd(TTA)_4_]^−^ anion crystallized with pyridinium (Leipoldt *et al.*, 1977 ▸), ammonium (Cary *et al.*, 2018 ▸) or a derivative of di­phenyl­iodo­nium (Chen *et al.*, 1997 ▸). The anion [*Ln*(TTA)_4_]^−^ has also been characterized with all other lanthanides, except with *Ln* = Ho and *Ln* = Lu. The coordination geometry is systematically close to square-anti­prismatic, with idealized point group *D*_4*d*_ (*e.g*. Assunção *et al.*, 2025 ▸; *Ln* = Eu). Ligands TTA^−^ are nearly planar: the dihedral angle between the thio­phene and *β*-diketonate moieties is 13.91 (16) or 5.30 (16)°. The angle formed between the planes of the six-membered chelate rings Nd1–O1–C1–C2–C3–O3 and Nd1–O11–C11–C12–C13–O13 in the asymmetric unit is 89.45 (7)°, indicating an almost orthogonal arrangement for the independent TTA^−^ ligands.

**Figure 2:**
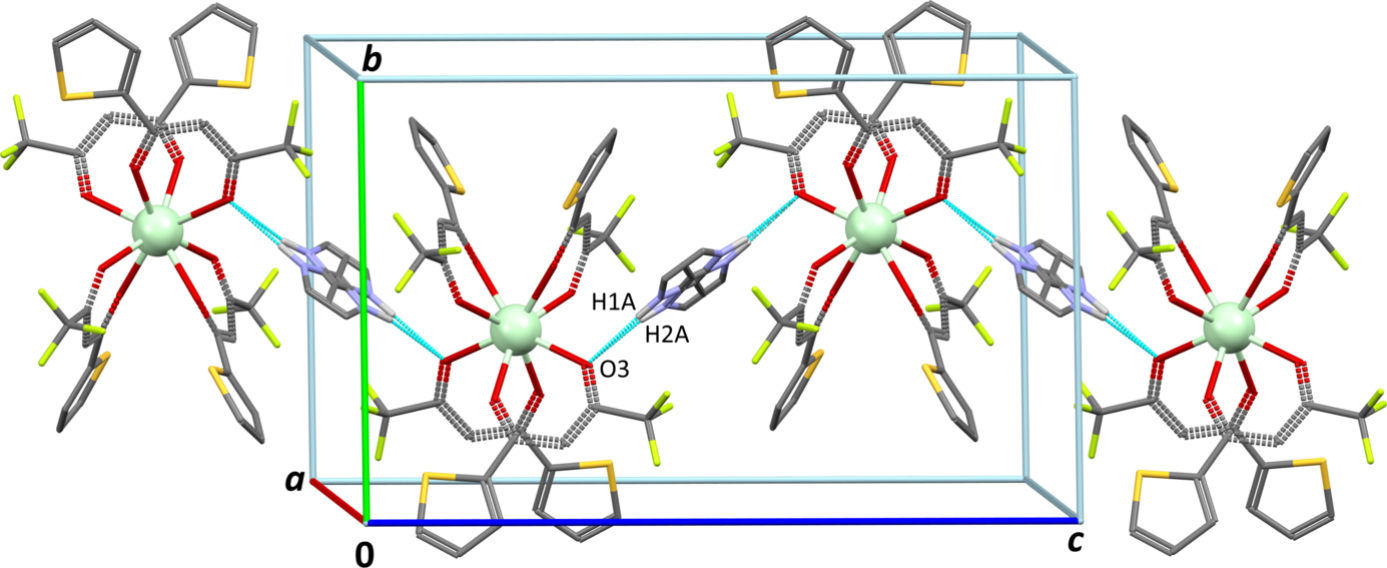
Part of the crystal structure, viewed nearly down [100]. One supra­molecular chain is shown, with N—H⋯O hydrogen bonds represented as blue dashed bonds. All H atoms not involved in hydrogen-bonding have been omitted for clarity.

Supra­molecular analysis of the title compound reveals inter­molecular contacts between anions and cations, involving NH groups in the cation as donors and O3 as acceptor (Table 1 ▸ and Fig. 2 ▸). Zigzag chains alternating cations and anions are formed, running along [001]. These chains are stacked in the crystal with no significant contacts. Since cations are then sandwiched by two thio­phene rings belonging to neighbouring anions along the chain, weak π–π inter­actions consolidate the crystal structure. In the asymmetric unit, the centroid-to-centroid separation between the thio­phene ring S2 and the imidazole ring is 4.212 (6) Å, and corresponding mean planes form a dihedral angle of 23.4 (6)°.

## Synthesis and crystallization

Crystals of [MeImH][Nd(TTA)_4_] were prepared *via* a sonochemical route using an Nd^3+^:HTTA = 1:2.5 stoichiometric ratio, starting from 1 mmol (0.591 g) of Nd(CF_3_SO_3_)_3_ dissolved in 25 ml of methanol and mixed with 25 ml of the ligand solution [2.5 mmol (0.555 g) of HTTA and 2 mmol (0.164 g) of MeIm] in deionized water. All solutions were stirred, mixed, and sonicated at room temperature using an Ultrasonic Processor equipment UP400St, applying a frequency of 24 kHz and power of 400 W with 1 pulsation per second for 20 min, until precipitation occurred. After a few hours at room temperature, the precipitate was separated from the mother liquor by centrifugation and washed with deionized water to remove soluble byproducts and/or excess of precursor materials. The dried product was kept at 343 K overnight, affording single crystals suitable for X-ray diffraction.

## Refinement

Crystal data, data collection and structure refinement details are summarized in Table 2 ▸. The cation [MeImH]^+^ is placed close to an inversion centre, and is thus equally disordered over two positions by symmetry. The occupancy for the cation in the asymmetric unit was fixed to 1/2. All H atoms were placed in idealized positions (Sheldrick, 2015*b* ▸).

## Supplementary Material

Crystal structure: contains datablock(s) I. DOI: 10.1107/S2414314625005693/wm4231sup1.cif

Structure factors: contains datablock(s) I. DOI: 10.1107/S2414314625005693/wm4231Isup2.hkl

CCDC reference: 2466617

Additional supporting information:  crystallographic information; 3D view; checkCIF report

## Figures and Tables

**Table 1:** Hydrogen-bond geometry (Å, °)

*D*—H⋯*A*	*D*—H	H⋯*A*	*D*⋯*A*	*D*—H⋯*A*
N1—H1*A*⋯O3^i^	0.86	2.03	2.865 (6)	163
N2—H2*A*⋯O3	0.86	2.06	2.904 (6)	168

Symmetry code: (i) 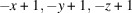.

**Table 2:** Experimental details

Crystal data	
Chemical formula	(C_4_H_7_N_2_)[Nd(C_8_H_4_F_3_O_2_S)_4_]
*M*_r_	1112.04
Crystal system, space group	Monoclinic, *C*2/*c*
Temperature (K)	295
*a*, *b*, *c* (Å)	17.3191 (4), 12.6547 (2), 20.5387 (4)
β (°)	103.772 (2)
*V* (Å^3^)	4372.01 (15)
*Z*	4
Radiation type	Ag *K*α, λ = 0.56083 Å
μ (mm^−1^)	0.78
Crystal size (mm)	0.31 × 0.26 × 0.24

Data collection	
Diffractometer	Stoe Stadivari
Absorption correction	Multi-scan (*X-AREA*; Stoe & Cie, 2019 ▸)
*T*_min_, *T*_max_	0.556, 1.000
No. of measured, independent and observed [*I* > 2σ(*I*)] reflections	97427, 8697, 7163
*R*_int_	0.043
(sin θ/λ)_max_ (Å^−1^)	0.782

Refinement	
*R*[*F*^2^ > 2σ(*F*^2^)], *wR*(*F*^2^), *S*	0.040, 0.113, 1.06
No. of reflections	8697
No. of parameters	313
H-atom treatment	H-atom parameters constrained
Δρ_max_, Δρ_min_ (e Å^−3^)	0.70, −0.90

Computer programs: *X-AREA* (Stoe & Cie, 2019 ▸), *SHELXT* (Sheldrick, 2015*a* ▸), *SHELXL* (Sheldrick, 2015*b* ▸), *Mercury* (Macrae *et al.*, 2020 ▸) and *publCIF* (Westrip, 2010 ▸).

## full crystallographic data

### 2-Methylimidazolium tetrakis(2-thenoyltrifluoroacetonato-κ2O,O')neodymium(III) Crystal data

**Table:**

(C_4_H_7_N_2_)[Nd(C_8_H_4_F_3_O_2_S)_4_]	*F*(000) = 2196
*M**_r_* = 1112.04	*D*_x_ = 1.689 Mg m^−^^3^
Monoclinic, *C*2/*c*	Ag *K*α radiation, λ = 0.56083 Å
*a* = 17.3191 (4) Å	Cell parameters from 122785 reflections
*b* = 12.6547 (2) Å	θ = 1.9–29.2°
*c* = 20.5387 (4) Å	µ = 0.78 mm^−^^1^
β = 103.772 (2)°	*T* = 295 K
*V* = 4372.01 (15) Å^3^	Irregular, blue
*Z* = 4	0.31 × 0.26 × 0.24 mm

### 2-Methylimidazolium tetrakis(2-thenoyltrifluoroacetonato-κ2O,O')neodymium(III) Data collection

**Table:**

Stoe Stadivari diffractometer	8697 independent reflections
Radiation source: Sealed X-ray tube, Axo Astix-f Microfocus source	7163 reflections with *I* > 2σ(*I*)
Graded multilayer mirror monochromator	*R*_int_ = 0.043
Detector resolution: 5.81 pixels mm^-1^	θ_max_ = 26.0°, θ_min_ = 1.9°
ω scans	*h* = −27→27
Absorption correction: multi-scan (X-AREA; Stoe & Cie, 2019)	*k* = −18→19
*T*_min_ = 0.556, *T*_max_ = 1.000	*l* = −32→32
97427 measured reflections	

### 2-Methylimidazolium tetrakis(2-thenoyltrifluoroacetonato-κ2O,O')neodymium(III) Refinement

**Table:**

Refinement on *F*^2^	Primary atom site location: dual
Least-squares matrix: full	Secondary atom site location: difference Fourier map
*R*[*F*^2^ > 2σ(*F*^2^)] = 0.040	Hydrogen site location: inferred from neighbouring sites
*wR*(*F*^2^) = 0.113	H-atom parameters constrained
*S* = 1.06	*w* = 1/[σ^2^(*F*_o_^2^) + (0.0519*P*)^2^ + 6.0925*P*] where *P* = (*F*_o_^2^ + 2*F*_c_^2^)/3
8697 reflections	(Δ/σ)_max_ = 0.001
313 parameters	Δρ_max_ = 0.70 e Å^−^^3^
0 restraints	Δρ_min_ = −0.90 e Å^−^^3^
0 constraints	

### 2-Methylimidazolium tetrakis(2-thenoyltrifluoroacetonato-κ2O,O')neodymium(III) Fractional atomic coordinates and isotropic or equivalent isotropic displacement parameters (Å^2^)

**Table:**

	*x*	*y*	*z*	*U*_iso_*/*U*_eq_	Occ. (<1)
Nd1	0.500000	0.38687 (2)	0.250000	0.03908 (6)	
S1	0.36020 (8)	0.07268 (11)	0.11099 (5)	0.0853 (3)	
O1	0.41250 (13)	0.23740 (15)	0.21178 (10)	0.0508 (4)	
C1	0.39885 (15)	0.1532 (2)	0.23872 (13)	0.0435 (5)	
F1	0.3888 (2)	0.2515 (3)	0.45231 (13)	0.1088 (10)	
C2	0.41016 (19)	0.1387 (2)	0.30859 (14)	0.0508 (6)	
H2	0.400999	0.072079	0.324391	0.061*	
F2	0.51077 (18)	0.2131 (2)	0.46517 (11)	0.0966 (8)	
S2	0.32140 (5)	0.68267 (7)	0.30984 (5)	0.0663 (2)	
F3	0.4226 (3)	0.0935 (2)	0.43956 (12)	0.1309 (15)	
O3	0.45289 (13)	0.31300 (15)	0.34528 (10)	0.0519 (4)	
C3	0.43400 (18)	0.2183 (2)	0.35402 (13)	0.0499 (6)	
C4	0.4388 (3)	0.1920 (3)	0.42796 (17)	0.0738 (11)	
F4	0.71200 (14)	0.6641 (2)	0.43086 (17)	0.1057 (10)	
C5	0.36846 (18)	0.0630 (2)	0.19456 (14)	0.0508 (6)	
F5	0.73633 (12)	0.50159 (18)	0.43148 (12)	0.0720 (5)	
F6	0.74347 (15)	0.5903 (4)	0.34738 (16)	0.1279 (14)	
C6	0.3428 (3)	−0.0367 (3)	0.21092 (18)	0.0749 (11)	
H6	0.342232	−0.058169	0.254091	0.090*	
C7	0.3182 (4)	−0.0996 (3)	0.1533 (3)	0.1059 (19)	
H7	0.298623	−0.167868	0.154069	0.127*	
C8	0.3258 (3)	−0.0518 (4)	0.0976 (2)	0.0946 (15)	
H8	0.313345	−0.083671	0.055615	0.114*	
C11	0.47677 (16)	0.61847 (19)	0.33016 (14)	0.0446 (5)	
O11	0.44749 (11)	0.53626 (15)	0.30053 (10)	0.0489 (4)	
C12	0.56012 (17)	0.6335 (2)	0.35716 (17)	0.0511 (6)	
H12	0.578136	0.699386	0.374301	0.061*	
C13	0.61449 (15)	0.5539 (2)	0.35866 (14)	0.0455 (5)	
O13	0.60391 (12)	0.46054 (15)	0.33646 (10)	0.0500 (4)	
C14	0.70171 (17)	0.5786 (3)	0.39209 (18)	0.0564 (7)	
C15	0.42182 (15)	0.7013 (2)	0.34063 (13)	0.0442 (5)	
C16	0.43723 (18)	0.7944 (2)	0.37833 (15)	0.0521 (6)	
H16	0.487658	0.819560	0.398658	0.063*	
C17	0.3646 (2)	0.8451 (3)	0.38110 (18)	0.0625 (8)	
H17	0.362778	0.907832	0.404259	0.075*	
C18	0.2991 (2)	0.7944 (3)	0.3473 (2)	0.0672 (9)	
H18	0.247536	0.817743	0.344799	0.081*	
N1	0.5374 (5)	0.5365 (5)	0.5489 (3)	0.0672 (17)	0.5
H1A	0.529815	0.579440	0.579218	0.081*	0.5
N2	0.5165 (6)	0.4413 (6)	0.4631 (3)	0.0714 (19)	0.5
H2A	0.492977	0.410787	0.426307	0.086*	0.5
C21	0.4825 (6)	0.5068 (19)	0.4964 (11)	0.070 (4)	0.5
C22	0.5920 (11)	0.4282 (12)	0.4934 (7)	0.079 (4)	0.5
H22	0.627250	0.383783	0.478873	0.094*	0.5
C23	0.6100 (10)	0.4887 (11)	0.5485 (6)	0.073 (3)	0.5
H23	0.658822	0.496792	0.578961	0.087*	0.5
C24	0.3995 (9)	0.5422 (14)	0.4791 (8)	0.097 (5)	0.5
H24A	0.365313	0.483663	0.482293	0.146*	0.5
H24B	0.386518	0.569207	0.434144	0.146*	0.5
H24C	0.392314	0.596802	0.509566	0.146*	0.5

### 2-Methylimidazolium tetrakis(2-thenoyltrifluoroacetonato-κ2O,O')neodymium(III) Atomic displacement parameters (Å^2^)

**Table:**

	*U*^11^	*U*^22^	*U*^33^	*U*^12^	*U*^13^	*U*^23^
Nd1	0.04478 (10)	0.03509 (8)	0.03947 (9)	0.000	0.01416 (7)	0.000
S1	0.1074 (8)	0.0947 (7)	0.0497 (4)	−0.0175 (6)	0.0106 (5)	−0.0124 (4)
O1	0.0579 (11)	0.0472 (9)	0.0457 (9)	−0.0091 (8)	0.0094 (8)	−0.0007 (7)
C1	0.0419 (11)	0.0438 (11)	0.0446 (12)	−0.0046 (9)	0.0095 (9)	−0.0045 (9)
F1	0.141 (3)	0.132 (2)	0.0769 (16)	−0.033 (2)	0.0713 (18)	−0.0225 (15)
C2	0.0627 (16)	0.0455 (12)	0.0450 (13)	−0.0166 (11)	0.0146 (12)	−0.0038 (9)
F2	0.117 (2)	0.116 (2)	0.0475 (11)	−0.0292 (17)	0.0014 (12)	0.0016 (12)
S2	0.0474 (4)	0.0646 (5)	0.0862 (6)	0.0010 (3)	0.0143 (4)	−0.0178 (4)
F3	0.254 (4)	0.0848 (16)	0.0529 (13)	−0.078 (2)	0.0341 (19)	0.0033 (11)
O3	0.0697 (12)	0.0464 (9)	0.0460 (9)	−0.0154 (8)	0.0265 (9)	−0.0084 (7)
C3	0.0585 (15)	0.0526 (13)	0.0419 (12)	−0.0170 (12)	0.0185 (11)	−0.0056 (10)
C4	0.108 (3)	0.070 (2)	0.0467 (15)	−0.036 (2)	0.0250 (18)	−0.0092 (14)
F4	0.0641 (13)	0.0690 (14)	0.160 (3)	0.0003 (11)	−0.0209 (15)	−0.0394 (16)
C5	0.0529 (14)	0.0501 (13)	0.0461 (13)	−0.0021 (11)	0.0051 (11)	−0.0091 (10)
F5	0.0547 (10)	0.0712 (12)	0.0823 (14)	0.0083 (9)	0.0007 (10)	0.0047 (10)
F6	0.0552 (13)	0.243 (4)	0.0871 (19)	−0.0286 (18)	0.0194 (13)	0.047 (2)
C6	0.113 (3)	0.0473 (15)	0.0555 (17)	−0.0206 (17)	0.0030 (19)	−0.0096 (13)
C7	0.153 (5)	0.060 (2)	0.089 (3)	−0.025 (3)	−0.002 (3)	−0.023 (2)
C8	0.104 (3)	0.093 (3)	0.072 (3)	−0.003 (3)	−0.007 (2)	−0.040 (2)
C11	0.0472 (12)	0.0408 (11)	0.0480 (12)	−0.0004 (9)	0.0154 (10)	−0.0020 (9)
O11	0.0450 (9)	0.0439 (9)	0.0598 (11)	−0.0013 (7)	0.0163 (8)	−0.0127 (8)
C12	0.0442 (12)	0.0396 (11)	0.0690 (17)	−0.0025 (9)	0.0123 (12)	−0.0091 (11)
C13	0.0429 (12)	0.0436 (11)	0.0504 (13)	−0.0022 (9)	0.0117 (10)	0.0001 (9)
O13	0.0503 (10)	0.0433 (9)	0.0548 (11)	0.0033 (7)	0.0089 (8)	−0.0053 (7)
C14	0.0434 (13)	0.0528 (14)	0.0712 (19)	−0.0011 (11)	0.0100 (13)	0.0008 (13)
C15	0.0433 (12)	0.0419 (11)	0.0485 (12)	0.0015 (9)	0.0133 (10)	−0.0030 (9)
C16	0.0554 (15)	0.0487 (13)	0.0519 (14)	0.0109 (11)	0.0123 (12)	−0.0075 (11)
C17	0.0682 (19)	0.0487 (14)	0.074 (2)	0.0105 (14)	0.0232 (16)	−0.0099 (14)
C18	0.0555 (17)	0.0618 (18)	0.088 (2)	0.0146 (14)	0.0246 (17)	−0.0015 (16)
N1	0.099 (5)	0.060 (3)	0.048 (3)	−0.021 (4)	0.029 (4)	−0.013 (2)
N2	0.108 (6)	0.066 (4)	0.048 (3)	−0.035 (4)	0.033 (4)	−0.017 (3)
C21	0.095 (11)	0.070 (6)	0.048 (5)	−0.032 (10)	0.026 (9)	−0.004 (4)
C22	0.104 (10)	0.066 (5)	0.077 (8)	−0.023 (5)	0.043 (8)	−0.013 (5)
C23	0.086 (7)	0.074 (6)	0.057 (5)	−0.027 (5)	0.017 (5)	−0.004 (4)
C24	0.084 (8)	0.113 (19)	0.091 (15)	−0.030 (11)	0.016 (12)	0.023 (9)

### 2-Methylimidazolium tetrakis(2-thenoyltrifluoroacetonato-κ2O,O')neodymium(III) Geometric parameters (Å, º)

**Table:**

Nd1—O13^i^	2.396 (2)	C7—H7	0.9300
Nd1—O13	2.396 (2)	C8—H8	0.9300
Nd1—O1^i^	2.4340 (19)	C11—O11	1.250 (3)
Nd1—O1	2.4340 (19)	C11—C12	1.430 (4)
Nd1—O11^i^	2.4346 (18)	C11—C15	1.466 (3)
Nd1—O11	2.4346 (17)	C12—C13	1.374 (4)
Nd1—O3	2.4758 (18)	C12—H12	0.9300
Nd1—O3^i^	2.4758 (18)	C13—O13	1.263 (3)
S1—C8	1.683 (5)	C13—C14	1.535 (4)
S1—C5	1.693 (3)	C15—C16	1.400 (4)
O1—C1	1.249 (3)	C16—C17	1.424 (4)
C1—C2	1.413 (4)	C16—H16	0.9300
C1—C5	1.475 (4)	C17—C18	1.345 (5)
F1—C4	1.330 (5)	C17—H17	0.9300
C2—C3	1.369 (4)	C18—H18	0.9300
C2—H2	0.9300	N1—C21	1.31 (2)
F2—C4	1.325 (5)	N1—C23	1.398 (19)
S2—C18	1.698 (4)	N1—H1A	0.8600
S2—C15	1.720 (3)	N2—C21	1.30 (2)
F3—C4	1.312 (4)	N2—C22	1.32 (2)
O3—C3	1.266 (3)	N2—H2A	0.8600
C3—C4	1.538 (4)	C21—C24	1.47 (2)
F4—C14	1.330 (4)	C22—C23	1.340 (13)
C5—C6	1.404 (4)	C22—H22	0.9300
F5—C14	1.316 (4)	C23—H23	0.9300
F6—C14	1.306 (4)	C24—H24A	0.9600
C6—C7	1.406 (5)	C24—H24B	0.9600
C6—H6	0.9300	C24—H24C	0.9600
C7—C8	1.327 (8)		
			
O13^i^—Nd1—O13	134.20 (9)	C6—C7—H7	123.4
O13^i^—Nd1—O1^i^	147.31 (7)	C7—C8—S1	112.9 (3)
O13—Nd1—O1^i^	76.30 (7)	C7—C8—H8	123.5
O13^i^—Nd1—O1	76.30 (7)	S1—C8—H8	123.5
O13—Nd1—O1	147.31 (7)	O11—C11—C12	123.6 (2)
O1^i^—Nd1—O1	78.00 (10)	O11—C11—C15	117.7 (2)
O13^i^—Nd1—O11^i^	70.90 (6)	C12—C11—C15	118.6 (2)
O13—Nd1—O11^i^	73.91 (7)	C11—O11—Nd1	134.77 (17)
O1^i^—Nd1—O11^i^	118.29 (7)	C13—C12—C11	122.3 (2)
O1—Nd1—O11^i^	137.14 (7)	C13—C12—H12	118.9
O13^i^—Nd1—O11	73.91 (7)	C11—C12—H12	118.9
O13—Nd1—O11	70.90 (6)	O13—C13—C12	129.6 (3)
O1^i^—Nd1—O11	137.15 (7)	O13—C13—C14	113.2 (2)
O1—Nd1—O11	118.28 (7)	C12—C13—C14	117.2 (2)
O11^i^—Nd1—O11	78.11 (9)	C13—O13—Nd1	130.54 (18)
O13^i^—Nd1—O3	113.71 (7)	F6—C14—F5	105.6 (3)
O13—Nd1—O3	83.79 (7)	F6—C14—F4	108.1 (3)
O1^i^—Nd1—O3	75.45 (7)	F5—C14—F4	105.0 (3)
O1—Nd1—O3	70.39 (6)	F6—C14—C13	111.0 (3)
O11^i^—Nd1—O3	148.98 (7)	F5—C14—C13	112.3 (3)
O11—Nd1—O3	74.30 (7)	F4—C14—C13	114.3 (3)
O13^i^—Nd1—O3^i^	83.79 (7)	C16—C15—C11	129.7 (3)
O13—Nd1—O3^i^	113.71 (7)	C16—C15—S2	111.2 (2)
O1^i^—Nd1—O3^i^	70.39 (6)	C11—C15—S2	118.89 (19)
O1—Nd1—O3^i^	75.45 (7)	C15—C16—C17	110.3 (3)
O11^i^—Nd1—O3^i^	74.30 (7)	C15—C16—H16	124.8
O11—Nd1—O3^i^	148.98 (7)	C17—C16—H16	124.8
O3—Nd1—O3^i^	135.63 (9)	C18—C17—C16	114.2 (3)
C8—S1—C5	92.2 (2)	C18—C17—H17	122.9
C1—O1—Nd1	133.56 (17)	C16—C17—H17	122.9
O1—C1—C2	124.5 (2)	C17—C18—S2	112.1 (2)
O1—C1—C5	117.7 (2)	C17—C18—H18	124.0
C2—C1—C5	117.8 (2)	S2—C18—H18	124.0
C3—C2—C1	122.9 (2)	C21—N1—C23	110.4 (10)
C3—C2—H2	118.6	C21—N1—H1A	124.8
C1—C2—H2	118.6	C23—N1—H1A	124.8
C18—S2—C15	92.21 (16)	C21—N2—C22	110.7 (9)
C3—O3—Nd1	127.90 (16)	C21—N2—H2A	124.6
O3—C3—C2	130.3 (3)	C22—N2—H2A	124.6
O3—C3—C4	112.9 (2)	N2—C21—N1	106.5 (10)
C2—C3—C4	116.8 (3)	N2—C21—C24	127.4 (17)
F3—C4—F2	107.4 (4)	N1—C21—C24	126.1 (18)
F3—C4—F1	106.5 (4)	N2—C22—C23	109.4 (17)
F2—C4—F1	106.0 (3)	N2—C22—H22	125.3
F3—C4—C3	114.9 (3)	C23—C22—H22	125.3
F2—C4—C3	110.4 (3)	C22—C23—N1	102.9 (15)
F1—C4—C3	111.2 (4)	C22—C23—H23	128.6
C6—C5—C1	129.4 (3)	N1—C23—H23	128.6
C6—C5—S1	110.7 (2)	C21—C24—H24A	109.5
C1—C5—S1	119.9 (2)	C21—C24—H24B	109.5
C5—C6—C7	110.9 (4)	H24A—C24—H24B	109.5
C5—C6—H6	124.5	C21—C24—H24C	109.5
C7—C6—H6	124.5	H24A—C24—H24C	109.5
C8—C7—C6	113.3 (4)	H24B—C24—H24C	109.5
C8—C7—H7	123.4		
			
Nd1—O1—C1—C2	25.1 (4)	C11—C12—C13—O13	2.6 (5)
Nd1—O1—C1—C5	−155.3 (2)	C11—C12—C13—C14	−177.1 (3)
O1—C1—C2—C3	3.2 (5)	C12—C13—O13—Nd1	23.9 (5)
C5—C1—C2—C3	−176.4 (3)	C14—C13—O13—Nd1	−156.4 (2)
Nd1—O3—C3—C2	−22.7 (5)	O13—C13—C14—F6	74.2 (4)
Nd1—O3—C3—C4	157.8 (2)	C12—C13—C14—F6	−106.0 (4)
C1—C2—C3—O3	−3.1 (6)	O13—C13—C14—F5	−43.7 (4)
C1—C2—C3—C4	176.4 (3)	C12—C13—C14—F5	136.0 (3)
O3—C3—C4—F3	−177.5 (4)	O13—C13—C14—F4	−163.1 (3)
C2—C3—C4—F3	2.9 (6)	C12—C13—C14—F4	16.6 (4)
O3—C3—C4—F2	−55.9 (4)	O11—C11—C15—C16	171.8 (3)
C2—C3—C4—F2	124.5 (3)	C12—C11—C15—C16	−5.0 (4)
O3—C3—C4—F1	61.5 (4)	O11—C11—C15—S2	−1.8 (3)
C2—C3—C4—F1	−118.1 (3)	C12—C11—C15—S2	−178.7 (2)
O1—C1—C5—C6	−174.7 (3)	C18—S2—C15—C16	−1.2 (2)
C2—C1—C5—C6	5.0 (5)	C18—S2—C15—C11	173.5 (2)
O1—C1—C5—S1	4.9 (4)	C11—C15—C16—C17	−172.8 (3)
C2—C1—C5—S1	−175.5 (2)	S2—C15—C16—C17	1.2 (3)
C8—S1—C5—C6	−1.1 (3)	C15—C16—C17—C18	−0.6 (4)
C8—S1—C5—C1	179.3 (3)	C16—C17—C18—S2	−0.3 (4)
C1—C5—C6—C7	179.9 (4)	C15—S2—C18—C17	0.9 (3)
S1—C5—C6—C7	0.3 (5)	C22—N2—C21—N1	0.3 (19)
C5—C6—C7—C8	0.9 (8)	C22—N2—C21—C24	179.8 (18)
C6—C7—C8—S1	−1.8 (8)	C23—N1—C21—N2	0.7 (18)
C5—S1—C8—C7	1.7 (5)	C23—N1—C21—C24	−178.7 (17)
C12—C11—O11—Nd1	−16.4 (4)	C21—N2—C22—C23	−1 (2)
C15—C11—O11—Nd1	166.90 (18)	N2—C22—C23—N1	1.6 (19)
O11—C11—C12—C13	−6.8 (5)	C21—N1—C23—C22	−1.5 (18)
C15—C11—C12—C13	169.9 (3)		

Symmetry code: (i) −*x*+1, *y*, −*z*+1/2.

### 2-Methylimidazolium tetrakis(2-thenoyltrifluoroacetonato-κ2O,O')neodymium(III) Hydrogen-bond geometry (Å, º)

**Table:**

*D*—H···*A*	*D*—H	H···*A*	*D*···*A*	*D*—H···*A*
C17—H17···F3^ii^	0.93	2.60	3.429 (4)	149
N1—H1*A*···O3^iii^	0.86	2.03	2.865 (6)	163
N2—H2*A*···O3	0.86	2.06	2.904 (6)	168
C24—H24*B*···S2	0.96	2.91	3.852 (15)	167

Symmetry codes: (ii) *x*, *y*+1, *z*; (iii) −*x*+1, −*y*+1, −*z*+1.
